# Calcineurin/NFAT signaling and innate host defence: a role for NOD1-mediated phagocytic functions

**DOI:** 10.1186/1478-811X-12-8

**Published:** 2014-01-30

**Authors:** Alain Vandewalle, Emilie Tourneur, Marcelle Bens, Cécilia Chassin, Catherine Werts

**Affiliations:** 1 Centre de Recherche sur l’Inflammation (CRI), UMRS 1149 et Groupe ATIP-AVENIR, Université Denis Diderot – Paris 7, Paris, France; 2 Institut Pasteur, Unité Biologie et génétique des parois bactériennes, Paris, France

**Keywords:** NFAT, Calcineurin inhibitors, NOD1, Bacterial phagocytosis

## Abstract

The calcineurin/nuclear factor of activated T cells (NFATs) signaling pathway plays a central role in T cell mediated adaptive immune responses, but a number of recent studies demonstrated that calcineurin/NFAT signaling also plays a key role in the control of the innate immune response by myeloid cells. Calcineurin inhibitors, such as cyclosporine A (CsA) and tacrolimus (FK506), are commonly used in organ transplantation to prevent graft rejection and in a variety of immune diseases. These immunosuppressive drugs have adverse effects and significantly increase host’s susceptibility towards bacterial or fungal infections. Recent studies highlighted the role of NFAT signaling in fungal infection and in the control of the pattern recognition receptor nucleotide-binding oligomerization domain-containing protein 1 (NOD1), which predominantly senses invasive Gram-negative bacteria and mediates neutrophil phagocytic functions. This review summarises some of the current knowledge concerning the role of NFAT signaling in the innate immune response and the recent advances on NFAT-dependent inhibition of NOD1-mediated innate immune response caused by CsA, which may contribute to sensitizing transplant recipients to bacterial infection.

## Introduction

Calcineurin inhibitors, such as cyclosporine A (CsA) and its newer counterpart FK506 (also called tacrolimus), are potent immunosuppressive drugs widely used to prevent acute graft rejection and in the treatment of a variety of autoimmune disorders. In addition to their potent inhibitory action on T cell receptor-mediated activation of the adaptive immune system, calcineurin inhibitors have many adverse side effects [1]. CsA, which is nephrotoxic, may induce chronic allograft nephropathy [2,3]. Long-term immunosuppressive treatment also favours the occurrence of fungal and bacterial infection [4-6]. Urinary tract infection (UTI), whether complicated by acute pyelonephritis (APN) or not, usually due to uropathogenic *Escherichia coli* (UPEC), represents the most frequent infectious complication after renal transplantation [4]. Although UTI and APN are generally considered to be relatively benign, a number of studies have suggested that they may increase the risk of graft loss and compromise long-term graft outcome [7,8]. Many factors contribute to the occurrence of post-transplantation UTI/APN. Combined effects of calcineurin inhibitors with lipopolysaccharide (LPS) endotoxins, rejection episodes, or recurrent UTIs may contribute to allograft injury during UTI/APN [9-11]. However, the mechanism(s) by which calcineurin inhibitors could directly modulate host-fungal or -bacterial interactions has remained largely unknown, until recent studies that have provided lines of evidence that the NFAT/calcineurin pathway, which interferes with the development of the myeloid lineage [12], plays important roles in the regulatory mechanisms of the immune innate system against pathogens.

The immune system recognizes a large variety of microorganisms and microbial-associated molecular patterns (MAMPs) through different pattern recognition receptors (PRRs) expressed by immune cells, such as polymorphonuclear neutrophils (PMNs), macrophages, natural killer (NK) cells, and dendritic cells (DCs), and also a variety of epithelial and non-epithelial cells [13,14]. The early recognition of MAMPs by PRRs, either present on the plasma membrane or in the cytosolic compartment, is essential for the removal of bacterial pathogens [15]. Once activated, PRRs initiate signaling cascades leading to the activation of the transcription factor NF-κB and mitogen-associated protein kinases (MAPKs). The subsequent production of pro-inflammatory mediators will then induce the activation and recruitment of immune cells, which play a key role in the first line of defence to kill invasive pathogens.

Several families of PRRs have been identified. They include Toll-like receptors (TLRs), NOD-like receptors (NLRs), RIG-like helicase (RLRs), and AIM2-like receptors (ALRs) [16-18]. TLRs are transmembrane receptors, which recognize a large variety of MAMPS in human and murine species. Among them, TLR2 forming heterodimers with TLR1 or TLR6 senses bacterial lipopeptides. TLR3 recognizes double-stranded RNA from viruses, TLR4 senses lipopolysaccharide from Gram-negative bacteria, TLR5 recognizes flagellin from flagellated bacteria, TLR7 recognizes single stranded RNA in endosomes, and TLR9 senses hypomethylated microbial DNA [19]. UPEC colonizing the urinary tract are recognized by several TLRs, including TLR2, 4, 5, 11, and perhaps 9 [20]. TLR11 is expressed in murine bladder epithelial cells and renal tubule cells, but is only represented by a pseudogene in humans [21]. Among NLRs, the nucleotide-binding oligomerization domain-containing protein 1 (NOD1) and 2 (NOD2) are two intracellular receptors that play important roles in the recognition of invasive pathogens [13,22].

Recently, interactions between the NFAT/calcineurin signaling pathways and PRRs have been established suggesting possible dysregulation of the innate immune response that could explain the greater susceptibility of transplanted patients treated with calcineurin inhibitors to fungal or bacterial infections. In addition, several groups have highlighted the role of the NOD1 receptor in the activation of neutrophil phagocytic functions against pathogenic bacteria, and shown that inhibition of NFAT/calcineurin signaling in myeloid cells could account for altered Nod1-mediated microbicide innate immune response.

This review will focus on the recent advances on the role of NFAT/calcineurin signaling and its interplay with NOD1-mediated phagocytic functions in the regulation of innate immune responses in myeloid cells, and on the adverse effects of calcineurin inhibitors on altered NFAT/calcineurin-dependent innate immune response, which may sensitize kidney grafts to fungal and bacterial infections.

### The NFAT/calcineurin signaling pathway and innate immunity

The Ca^2+^/calmodulin/calcineurin pathway regulates the activity of the transcription factors of the nuclear factor of activated T cells (NFAT) family. The NFAT family encompasses five individually encoded members. NFAT1 (also called NFATc2 or NFATp), NFAT2 (NFATc1 or NFATc), NFAT3 (NFATc4), NFAT4 (NFATc3 or NFATx) [23], and NFAT5 (also called TonEBP or OREBP) [24]. All NFATs share a similar DNA-binding domain, and are modulated by calcineurin, a calcium, calmodulin-dependent serine/threonine protein phosphatase, consisting of a catalytic subunit calcineurin A (CnA), and a tightly associated Ca^2+^-binding subunit, calcineurin B (CnB) [25]. NFAT5, which differs in its structure from the other NFATs, is not regulated by calcium, but is activated in response to osmotic stress. NFATs are maintained in an inactive state in the cytosol of resting cells. Upon the stimulation of intracellular Ca^2+^ influx, calmodulin is activated and dephosphorylates the phosphorylation motifs from the N-terminus of NFATs, allowing NFATs to translocate to the nucleus where they collaborate with other transcription factors, such as AP-1, to induce gene transcription [26]. Calcineurin inhibitors, which inhibit the phosphatase activity of calcineurin and the nuclear translocation of NFATs, are currently used to prevent graft rejection in transplant recipients. Impaired activation of NFATs will prevent the transcription of cytokine genes, including IL-2, in activated T cells [26]. However, in addition to their major functions in lymphocytes in adaptive immunity, new roles of NFATs in innate immunity have been identified in myeloid cells [27].

Dectin-1, which plays a key role in the recognition of pathogenic fungi, is a β-glucan receptor belonging to the C-type lectin receptors, and is activated by zymosan, a cell-wall constituent of *Candida* species [28-30]. Upon ligand activation, dectin-1 cooperates with TLR2 to stimulate NF-κB and regulate cytokine production [31]. Dectin-1 alone was shown to induce phagocytosis and Src and Syk kinases-mediated induction of reactive oxygen species (ROS) via the activation of NFAT in macrophages and DCs [32]. Dectin-1 also regulates the induction of members of early growth response (Egr) family, cyclooxygenase-2 (COX2), and IL-2, IL-10, and IL-12 p70 productions by DCs stimulated by zymosan [32]. CD14 was also shown to induce Ca^2+^ influx and NFAT activation causing apoptosis of differentiated DCs, but not macrophages, stimulated by LPS [33]. NFAT2/c1 activated by the receptor activator of NF-κB (RANKL) was also shown to play a key regulatory role in the terminal differentiation of osteoclasts [34].

Recently, Fric et al. [12] demonstrated that NFAT1/c2 acts as a negative regulator of myeloid lineage development. These authors showed that inhibition of calcineurin/NFAT signaling increases the number of myeloid progenitors, and that the two calcineurin inhibitors CsA and FK506 antagonized the development of DCs induced by the Fms-related tyrosine kinase 3 ligand (Flt3-L). These findings demonstrated that calcineurin/NFAT signaling contributes to maintenance of innate immune homeostasis [35].

A number of studies have provided indirect and more direct evidence that NFAT/calcineurin pathways interfere with the regulatory mechanisms of innate immune defences. Calcineurin inhibitors or knockdown of calcineurin mRNA expression activate NF-κB and TLR-mediated MAPK pathways in macrophages, while over-expression of a constitutively activated form of the CnA subunit inhibits TLR- activated pathways [36]. The development of selective inhibitors of NFAT, such as VIVIT, a high-affinity calcineurin-binding peptide selected from combinatorial peptide libraries based on the calcineurin docking site of NFAT [37], and the more recent cell permeable inhibitor of NFAT, 11R-VIVIT [38] have proved to be useful tools to analyse in vivo the functions of NFAT. 11R-VIVIT and FK506 significantly inhibit LPS and LPS plus IFN-γ induced IL-12 expression independently of IL-10 in macrophages, whereas in vivo administration of 11R-VIVIT was shown to significantly improve inflammatory lesions in an experimental model of colitis [39]. The leucine-rich repeat kinase 2 (LRRK2), identified as a major susceptibility gene for Crohn’s disease [40,41], was reported to act as a negative regulator of NFAT1/c2-induced cytokine responses. LRRK2 modulates the cytoplasm retention of NFAT and the interaction between NFAT1 and the non coding RNA NFAT repressor (NRON) complex [42] in response to inducer of the innate immunity [43]. Furthermore, severe experimental colitis induced by dextran sulphate sodium (DSS) in LRRK2 deficient (*Lrrk2*^*-/-*^) mice was associated with enhanced nuclear localization of NFAT1. VIVIT was also shown to inhibit TNF-α induced expression in mouse bone marrow macrophages (BMMs) stimulated by the TLR4 ligand, LPS, and the TLR1/2 ligand, Pam3CSK4. In addition, LPS stimulation did not induce the nuclear translocation of NFAT1/c2 and NFAT2/c1, but in contrast, BMMs exhibited constitutive nuclear localization of NFAT4/c3 and NFAT3/c4, regardless of LPS stimulation. Moreover, VIVIT removed NFAT4 and NFAT3 from the nucleus and inhibited TLR-mediated activation of TNF [44]. Overall, these findings have highlighted regulatory roles of NFATs on different aspects of the immune response, and suggest that the NFATs may have distinct functions according to the cell type or pathogen considered.

**Figure 1:**
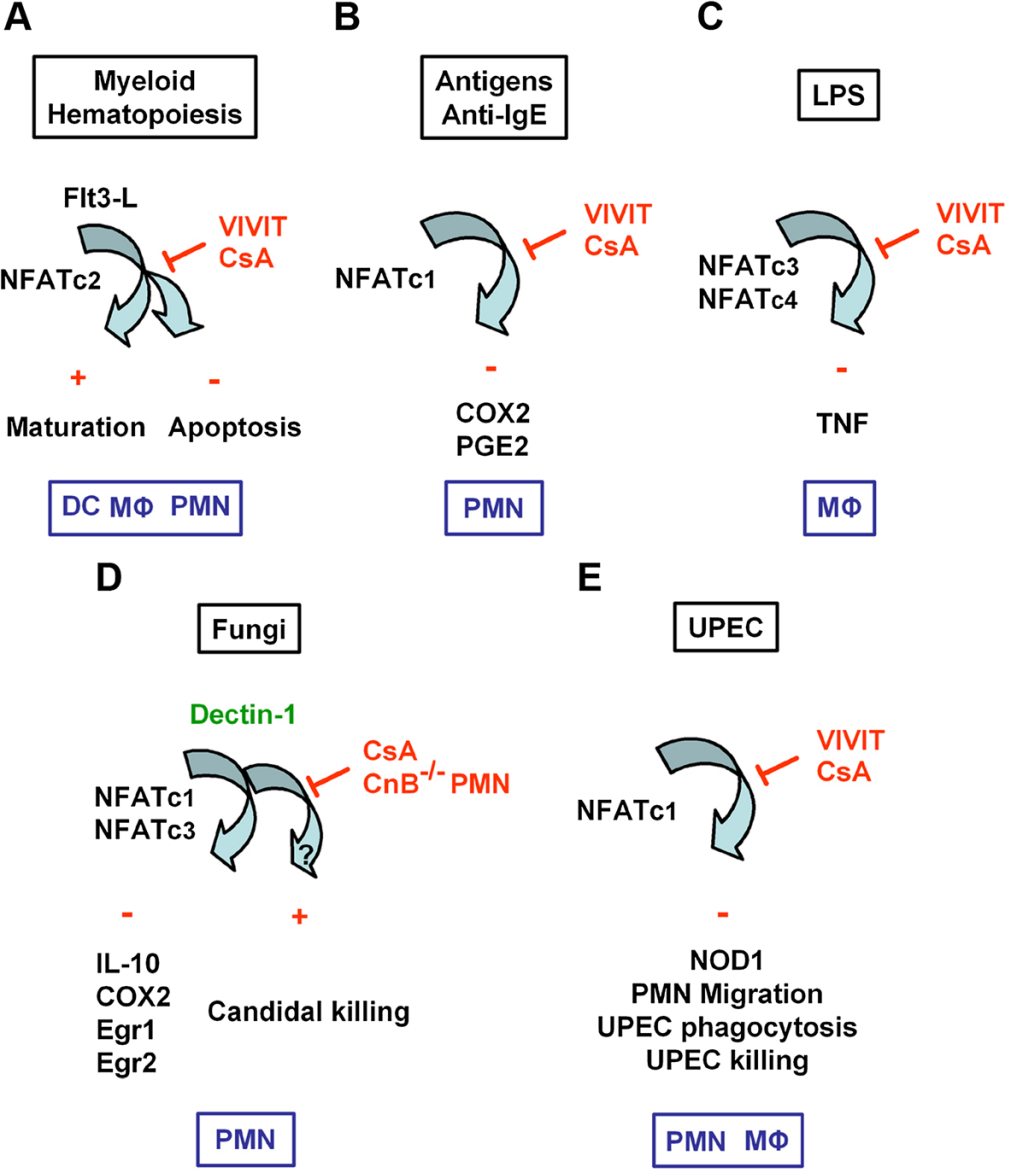
**Differential activation of NFATc in myeloid cells.** Schematic representation of the activation and inhibition of NFATc by calcineurin inhibitors in myeloid cells. **(A)** Inhibition of calcineurin/NFAT signaling by cyclosporine A (CsA) or the NFAT peptide inhibitor, VIVIT, antagonizes Flt3-L-induced development of bone marrow myeloid cells and increases the number and proliferation of myeloid progenitors [12]**(B)** Antigens- and anti-IgE-dependent activation of NFAT2/c1 and up-regulation of COX2 expression and release of PGE2. CsA and the peptide inhibitor of NFAT, VIVIT, both inhibited antigens- and anti-IgE-mediated activation of COX2 and PGE2 in human neutrophils [45]. (**C)** LPS activates NFAT3/c4 and NFAT4/c3 in mouse macrophages, and CsA and VIVIT both induced significant inhibition of the LPS-induced TNF production [44]. **(D)** Zymosan and curdlan failed to activate IL-10, COX2, Egr1 and Egr2 expressions regulated by NFAT2/c1 and NFAT4/c3 in CsA-treated and CnB-deficient neutrophils, indicating that the dectin-1 receptor is the upstream activator of calcineurin. *C. albicans* killing was not affected in NFAT-deficient neutrophils, suggesting that the CnB regulation of antifungal response may occur through an NFAT-independent anti-microbial mechanism [46]. **(E)** Downexpression of the NFAT2/c1 by silencing RNA (siRNA) impaired the activation of NOD1 induced by UPEC. CsA or the cell permeable 11R-VIVIT inhibited the UPEC-induced NOD1 expression and NOD1-mediated neutrophil functions (migration capacity, phagocytosis, bacterial killing) [47]. DC: Dendritic cell; MΦ: Macrophage; PMN: Polymorphonuclear neutrophil.

Although the regulatory role of NFAT1 and other NFATs has been extensively studied in myeloid cells, only a few studies have analysed the expression and the role of NFAT/calcineurin signaling in neutrophils. Vega et al. [45] first reported the expression of NFAT2/c1 in human PMNs. Antigens, anti-IgE, and anti-FcϵR induced Ca^2+^ stimulation, which increases cellular calcineurin activity and the nuclear translocation of NFAT2/c1, while CsA and VIVIT abolished antigens- and anti-IgE-induced cyclooxygenase-2 (COX2) upregulation and prostaglandin E2 (PGE2) release [45]. Greenblatt et al. [46] also evidenced NFAT2/c1 and NFAT4/c3 expression in murine PMNs, and showed that mice with a conditional deletion of CnB in neutrophils, like CsA-treated mice, failed to control *C. albicans* infection without affecting the classical fungicidal activity, including ROS production and phagocytosis in response to *C. albicans* or zymosan stimulation. However, both CsA-treated neutrophils and CnB-deficient neutrophils exhibited impaired production of IL-10 after stimulation by zymosan and curdlan, which is the specific dectin-ligand with no TLR2 and TLR4 stimulating properties [46]. Altogether, these findings evidenced a novel, not yet fully characterized, NFAT-dependent and -independent candidacidal mechanism beyond dectin-1 that could account for the disseminated fungal infections observed in CsA-treated patients, independently of the effect of the immunosuppressive drugs on the adaptive immune response [46]. Recently, we also showed that knockdown of *NFAT2/c1* mRNA expression by silencing mRNAs, similar to the inhibitory action of the 11R-VIVIT on NFATs, markedly inhibited the *Nod1* mRNA expression without affecting *Tlr2* and *Tlr4* mRNA expressions in mouse macrophages activated by UPEC [47]. In vivo administration of CsA or FK506 (AV, ET, MB, CC, and CW, unpublished results), or 11R-VIVIT also markedly impaired the neutrophil bacterial phagocytic killing capacity of UPEC and increased the renal susceptibility to UPEC using an experimental murine model of ascending UTI [47]. These findings indicate that CsA may directly alter NOD1 expression and *E. coli* killing capacity by neutrophils. In line with these findings, a number of recent studies have provided evidence that NOD1 plays an important role in the regulation of neutrophil phagocytic function, which represents the main first line of defence against pathogenic bacteria. Figure 1 illustrates the participation of NFATc in the activation of the innate immune response in myeloid cells in response to various effectors. The following paragraphs will summarise the current knowledge of how NOD1 takes part in the regulation of the immune innate response to invasive pathogenic bacteria.

### NOD1 signaling

NOD1 (CARD4) belongs to the NLR family of multi-domain protein, including NOD2 (CARD15), and consists of an amino-terminal caspase activation and recruitment domain (CARD), a nucleotide-binding oligomerization domain (NBD), and a C-terminal leucine-rich repeat domain (LRR) [48,49], that is also found in TLRs and that has been linked to resistance to infections [48]. The binding of MAMPs to the LRR domain results in the activation of signaling through homophilic CARD-CARD interactions [49,50]. NOD1 contains a single CARD domain, while NOD2 contains two domains [51]. NOD1 and NOD2 are two intracellular receptors that recognize bacterial peptidoglycan fragments. NOD1 recognizes γ-D-glutamyl-meso-diaminopimelic acid (meso-DAP), a degradation product of peptidoglycan (PGN) containing DAP [52,53], which is present in most Gram-negative bacteria, such as *Shigella flexneri*, enteroinvasive and uropathogenic *E. coli*, *Chlamydia*, or *Pseudomonas aeruginosa*[47,54-57], *Helicobacter pylori*[58], and some Gram-positive bacteria [59,60]. In contrast, NOD2 recognizes muramyl dipeptide (MDP), a motif common to PGNs from all classes of bacteria [61]. NOD1 is ubiquitously expressed, while NOD2 is mainly found in macrophages, DCs, Paneth cells, and a variety of epithelial cells [13]. Mutations in the *CARD15* gene encoding NOD2 have been shown to be associated with Crohn’s disease, a chronic inflammatory bowel disease mainly driven by T cells [62,63].

**Figure 2:**
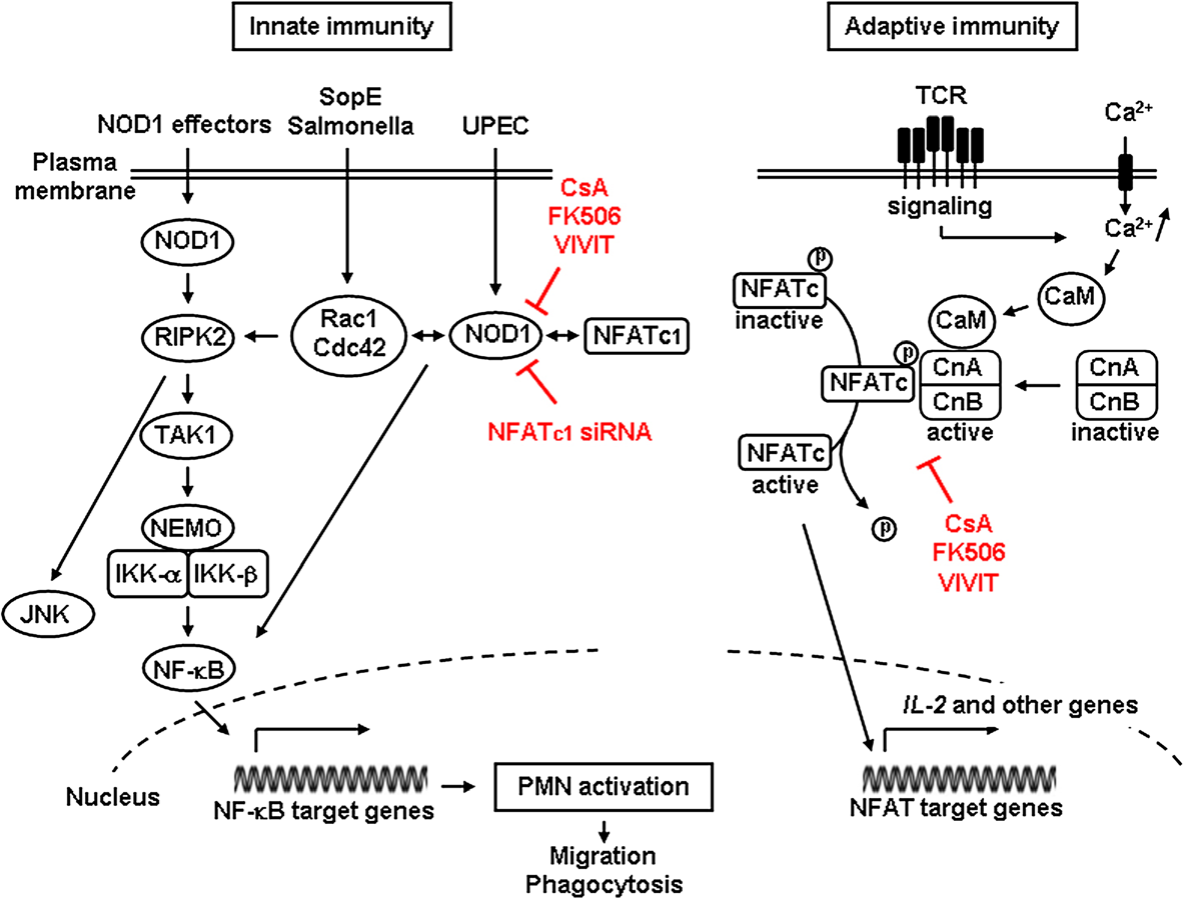
**NOD1 signaling and interactions with Rho GTPases and NFATc.** (Left panel) Schematic representation of the activation of NOD1 interacting with Caspase recruitment domain (CARD)-containing kinase RIPK2, which leads to activation of RIPK2 and subsequent recruitment and activation of TAK1. The TAK1 complex then induces the polyubiquitination of the IKK-β kinase, degradation of the NF-κB repressor IκB, nuclear translocation of NF-Kb, and transcription of pro-inflammatory mediators. NOD1 stimulating agonists also activate JNK. The guanine exchanger SopE from salmonella activates Rho GTPase Rac1 and Cdc42, which form a protein complex with NOD1 and the heat shock protein 90 (not shown). Activation of Rac1 and Cdc42 induces the activation of NOD1/RIPK2 signaling and induction of NF-κB-mediated pro-inflammatory mediators. Downregulation of NOD1 expression by calcineurin inhibitors or silencing NFATc1 mRNA markedly impairs bacterial-mediated activation of PMN functions (e.g. neutrophil migration and *E. coli* phagocytic killing capacities). (Right panel) Schematic view of the NFATc activation pathway by cell surface receptors coupled to Ca^2+^ mobilization. Ca^2+^-dependent activation of calmodulin (CaM) and calcineurin B (CnB) enables CaM binding to CnA regulatory region, and CnA activating conformational change. Activated calcineurin then dephosphorylates the phosphorylation motifs of NFATc, allowing NFATc to translocate to the nucleus. Nuclear NFATc, in collaboration with other transcription factors (such as AP-1), then induce gene transcription. Calcineurin activity is inhibited by CsA or FK506, which binds to their intracellular immunophillin receptors. The high-affinity calcineurin-binding VIVIT peptides are potent blockers of NFATc. IKK: I kappa B kinase; JNK: c-Jun N-terminal kinases; NEMO: NF-κB essential modulator; NF-κB: nuclear factor κB; RIPK2: Receptor-interacting serine/threonine-protein kinase 2; TAK1: Transforming growth factor-beta-activated kinase 1; TCR: T cell receptor.

Upon ligand recognition, the NBD domain of NOD1 or NOD2 oligomerizes and initiates the interaction of the CARD domain with RIPK2 (also called RIP2/RICK), which is a member of the CARD protein family [49,55]. RIPK2 is activated subsequently by proximity and promotes the formation of a signaling complex that contains the regulatory subunit of the IKK complex, NEMO [64], leading to NF-κB activation (see Figure 2, left panel). NOD1 also induces the activation of *c-*Jun NH2*-*terminal kinase (JNK) pathway [55,65] and apoptosis [66]. NOD1 activating ligands were shown to enter cells through an endocytic process, most likely in a clathrin-dependent manner, and the cytosolic internalization of NOD1 ligands is pH-dependent [67]. NOD1 was also shown to be localized in the cytosol and plasma membrane of human intestinal cells, and imaging studies revealed that NOD1 is recruited to the site of entry of invasive *S. flexneri*[68].

### NOD1 regulates neutrophil phagocytic functions

The rapid production of chemokines by immune cells and epithelial cells induces the rapid recruitment of polymorphonuclear neutrophils (PMNs) to the site of inflammation, which represent the first line of defence of the innate immune system against extracellular pathogens [69]. Masumoto et al. [70] first reported that the administration of the synthetic NOD1 ligand KF1B in WT mice induced the rapid production of CCL2/MCP1 and CXCL2/MIP-2 and the recruitment of intraperitoneal PMNs, but not lymphocytes and macrophages. This effect appeared to be NOD1-dependent since the recruitment of PMNs induced by the active NOD1-stimulatory compound KF1 was abolished in NOD1 deficient (*Nod1*^*-/-*^*)* mice. Dharancy et al. [71] also showed that upon liver injury induced by carbon tetrachloride (CCL4) intoxication, the in vitro migration capacity of PMNs induced by chemoattractants chemokines or formyl-methionyl-leucyl-phenylalanine (fMLP) was significantly reduced in *Nod1*^*-/-*^ PMNs compared to WT PMNs. These authors also showed that the number of infiltrating PMNs was lower in the liver from *Nod1*^*-/-*^ mice than WT mice subjected to ischemia-reperfusion injury. Conversely, FK565, another potent synthetic NOD1 agonist [72], significantly stimulated the migration of PMNs [71]. *Nod1*^*-/-*^ mice were also shown to exhibit impaired production of CXCL1 and defective recruitment of neutrophils to the intestine after *Clostridium difficile* infection, suggesting that NOD1-mediated neutrophil recruitment regulates susceptibility towards *C. difficile* in the intestine [60]. We also showed using a murine model of ascending UTI that *Nod1*^*-/-*^, but not *Nod2*^*-/-*^ mice, were more susceptible than WT mice to the retrograde inoculation of UPEC, and exhibited impaired recruitment of neutrophils in the UPEC-infected kidneys [47].

The process governing PMN extravasation from blood vessels involves a complex multistep cascade that is orchestrated by a tightly coordinated sequence of adhesive interactions with vessel wall endothelial cells. The recruitment of PMNs involves a cascade of adhesive and migratory events, including the capture and selectin-mediated rolling of PMNs along the vessels, chemokine-induced activation of PMNs, and integrin-dependent adhesion and subsequent trans-endothelial migration [73-76]. Surface expression levels of the β2-integrin CD11b/CD18 decreased from about 50% in liver PMNs from CCL4-treated *Nod1*^*-/-*^ mice compared to that of their WT counterparts, and fMLP failed to activate CD11b expression in *Nod1*^*-/-*^ neutrophils [71]. In contrast, the synthetic agonist FK565 increased β2-integrin expression in neutrophils infiltrating injured liver after CCL4 intoxication. Renal bacterial loads were also significantly greater in the infected kidneys from *Nod1*^*-/-*^ mice than from their WT counterparts, 24 h after the transurethral inoculation of UPEC [47]. Like that occurring in *Nod1*^*-/-*^ livers subjected to ischemia [71], the number of GR1^high+^, CD11b^high+^ neutrophils was also significantly less in kidneys from infected *Nod1*^*-/-*^ mice than in WT mice, 24 h after UPEC infection [47].

The Weiser group demonstrated that NOD1 is involved in the phagocytic killing capacity of bone marrow-derived neutrophils [77]. This group showed, using a mouse model of airway bacterial co-infection with the Gram-positive pathogen *Streptococcus pneumoniae* and Gram-negative Haemophilus influenzae (*Hi*), that neutrophils from mice treated with the Gram-negative bacteria *H. influenzae* containing synthetic PGN fragments containing meso-DAP, activate cytoplasmic NOD1 and facilitate neutrophil opsonophagocytic killing of the Gram-positive *Streptococcus pneumoniae*[78]. Clarke et al. [79] then demonstrated that PGN can translocate from the gut into bone marrow cells, and that in vivo administration of a synthetic NOD1 ligand, but not the NOD2 ligand MDP, can restore neutrophil phagocytic functions after antibiotic-induced microbiota depletion [79]. These authors also showed that *Nod1*^*-/-*^ mice were more susceptible than WT mice to *S. pneumoniae* infection. Overall, these findings have provided clear evidence that PGN, through NOD1, can stimulate innate immunity in mouse neutrophils. These results are relevant to humans, since we found, ex vivo, in PMNs of human transplant patients a downregulation of *NOD1* mRNA associated with reduced *E. coli* phagocytosis properties [47].

### NOD1 interacts with Rho GTPases to stimulate the innate immune response

The mechanism by which NOD1 stimulates bacterial phagocytosis has been first attributed to a priming effect of the immune system [79], but recent studies evidenced close interplay between NOD1 and the small Rho GTPases [80]. Activated Rho GTPases Rac1 and Rac2, and Cdc42 [81,82], which lead to rapid actin rearrangements, play key roles in the activation of phagocytic processes [83]. Rac1 was shown to regulate PGN activation of the NF-κB signaling pathway through the recruitment of the p85 regulatory phosphoinositide 3-kinase (PI3K) subunit in macrophages [84]. Pathogenic bacteria also synthesize a number of virulent factors activating or mimicking small Rho GTPase proteins in host cells [85]. Most of the factors activating Rho GTPases have been identified in Gram-negative bacteria, such as the cytotoxic necrotizing Factor 1 (CNF1) expressed in UPEC strains, or the *Salmonella* outer protein E (SopE/SopE2) from *S. Typhimurium*[86]. These factors were shown to stimulate the innate immune response. For example, SopE/SopE2 and SopB from *S. Typhimurium* stimulate Rho GTPases leading to NF-κB and MAPKs activation [87]. The guanine nucleotide exchange factor H1 (GEF-H1) from *S. flexneri*, which interacts with NOD1, was shown to be required for the RIK2-dependent activation of NF-κB [88]. Boyer et al. [89] also showed that the *E. coli* cytotoxic necrotizing factor 1 (CNF1), the prototypal bacterial toxin activating host GTPases, activates Rac2, which then interacts with the innate immune adaptors IMD (the fly ortholog of RIP1/RIPK2), and RIP1 and RIPK2 to induce NF-κB activation and IL-8 expression in mammalian cells. Keestra et al. [90] using a mouse model of *S. Typhimurium* infection and transfected HEK293 cells, have shown that the activation of Rac1 and Cdc42 by bacterial delivery or SopE expression stimulate NOD1 signaling and downstream RIPK2-mediated stimulation of the NF-κB inflammatory response. These authors also showed that PGN detects NOD1 by sensing the activation of Rac1. These findings, which are summarised in the left panel from Figure 2, have provided the first direct demonstration that pathogen-induced NOD1 signaling requires small Rho GTPases.

### Cyclosporine A impairs NFAT/NOD1-mediated renal antibacterial defence

The impact of immunosuppressive therapy, which increases susceptibility towards bacterial infection has been considered for a long time to be largely non specific. However, former studies have provided indirect evidence that calcineurin inhibitors may affect neutrophil functions [91-94]. The decreased renal susceptibility to UPEC following CsA treatment has been attributed to NFATc1-dependent inhibition of NOD1-mediated innate immune response [47]. Figure 2 summarises the main sites of inhibitory action of CsA and FK506, and VIVIT peptides on the Ca^2+^-dependent calcineurin/NFAT signaling leading to the activation of NFAT activated genes, such as IL-2. The inhibitory effects of these agents on NOD1 signaling pathway and subsequent activation of neutrophil functions are also shown.

CsA may differently affect immune receptors. CsA, which inhibits *Nod1* mRNA expression in myeloid cells, also blunts *Tlr4* mRNA expression without affecting *Nod1* and *Nod2* expression in renal tubule cells, suggesting that the combined decrease in TLR4 mRNA and protein expression in renal tubule cells and in NOD1 in myeloid cells caused by CsA should contribute to the observed decreased resistance to UPEC [47]. Immunosuppressant therapy increases the susceptibility towards bacterial and viral infection, which explains the incidence of infectious events in solid organ recipients. However, the concentrations of calcineurin inhibitors generally used in vitro are generally about 10-fold higher than those used in humans to prevent activation of lymphoid cells. The question arises as to whether or not the concentration of calcineurin inhibitors used during current immunosuppressive regimens are sufficient to fully suppress NFATc in myeloid cells. We reported that incubating murine myeloid cells with a concentration of 100 nM (~ 120 ng/ml) CsA, which is in the same range as the serum concentrations of CsA found in renal transplant recipients, markedly downregulated the expression of NOD1 (but not of TLR2 and TLR4). Clinical studies also evidenced a similar decrease in NOD1 mRNA and functional response in leukocytes from transplant recipients treated with CsA [47]. These findings suggest that low concentrations of CsA can be sufficient to impair NFAT activation and/or nuclear residence. By contrast to the observation that FK506 can induce reduced responsiveness to LPS in DCs and macrophages [95], Tourneur et al. [47] did not evidence marked decrease in either LPS- or Pam3CSK4-induced IL-8 production in intact leukocytes from transplant recipients receiving CsA. Given that interplays between NODs and TLRs can be critical for the induction of protective immune responses [52,96], it cannot be excluded that the possible induction of TLR4 tolerance together with decreased NOD1-mediated phagocytic functions caused by calcineurin inhibitors contribute to impaired resistance of transplant recipients to UPEC colonizing renal grafts. A better understanding of the mechanism of activation of small Rho GTPases and NOD1 by virulent effectors produced by pathogens should provide new insights in the mechanisms triggering bacterial phagocytosis.

## Conclusion

A number of studies have recently defined new roles in the regulation by calcineurin/NFAT signaling of the innate immune system in myeloid cells and provided a better understanding of the altered immune response caused by calcineurin inhibitors relevant to the frequency of disseminated fungal infection and UTI/APN seen in renal transplant recipients. The consequence of activating NFAT signaling may differ depending on the cell type and effectors considered. Although our understanding of the regulation of NFAT signaling has been greatly improved, the redundant role of the different NFATc proteins still renders difficult in vivo studies on the exact function of the different NFAT signaling pathways. The development of pharmacological strategies aimed at specifically inhibiting NFAT activation, such as VIVIT peptides, also remains limited and should deserve further studies to assess the use of such peptide inhibitors in vivo.

Given that calcineurin inhibitors that interact with NFAT are still used in the prevention of graft rejection and in the treatment of chronic autoimmune disorders, the development of new strategies aimed to reduce the occurrence of bacterial or fungal infection still remains justified. Because CsA and FK506 remain indispensable for preventing transplant rejection, different strategies have been developed to decrease their adverse effects by reducing the concentrations of calcineurin inhibitors alone or in combination with other suppressive drugs [mycophenolate mofetyl (Cellcept), rapamycin (a potent mTOR inhibitor) or monoclonal antibodies [97]. As also stated by Zanoni and Granucci [98], a better understanding of the specific role of the NFATc proteins differently expressed in immune cells, and the development of new tools allowing more specific in vivo inhibition of the different NFATc should provide a basis for the development of drugs with more specific actions than CsA or FK506.

A number of studies have provided convincing evidence that pre-treatment of mice with NOD agonists enhances host protection against sepsis, bacterial infection, viruses, or even parasites [99]. Given that NOD1 synthetic agonists can restore NOD1-mediated host protective functions in CsA-treated mice [47], the administration of synthetic NOD1 agonists alone or in combination with antibiotics might be potentially helpful to reduce the occurrence of ascending UTI and APN in renal graft. The immunoprotective effect of the NOD1 agonists, which restored the renal defence of CsA-treated mice against invasive UPEC [47], certainly results from their immunostimulatory properties, which enhance host protective functions by increasing the levels of protective factors. However, the exact mechanism of NOD1 activation by synthetic NOD1 agonists remains largely unknown. In addition, further studies should be required to determine the timing of prophylactic effects provided by NOD1 ligands on neutrophil functions to avoid blunting of their innate immune capacities.

## Abbreviations

AP-1: Activator protein 1; APN: Acute pyelonephritis; CARD: Caspase activation and recruitment domain; CsA: Cyclosporine A; Cylclooxygenase-2: COX2; JNK: c-Jun N-terminal kinase; MAPK: Mitogen-activated protein kinase; MAMP: Microbial-associated molecular pattern; NFAT: Nuclear factor of activated T cells; NF-κB: Nuclear factor kappa B; NLR: NOD-like receptor; NOD1: Nucleotide-binding oligomerization domain-containing protein 1; NOD2: Nucleotide-binding oligomerization domain-containing protein 2; PI3K: Phosphoinositide 3-kinase; PMN: Polymorphonuclear neutrophil; PRR: Pattern recognition receptor; RIPK2: Receptor-interacting serine/threonine protein kinase 2; ROS: Reactive oxygen species; TLR: Toll-like receptor; UPEC: Uropathogenic *E. coli*; UTI: Urinary tract infection.

## Competing interests

The authors declare that they have no competing interests.

## Authors’ contributions

ET, MB, and CC participated in the design of the study. AV and CW conceived and wrote the manuscript. All authors read and approved the final manuscript.
